# A case report navigating CVID and sarcoidosis overlaps in pediatric nephritis

**DOI:** 10.3389/fped.2024.1417724

**Published:** 2024-09-18

**Authors:** Amanda Salih, Amanda Brown, Amanda Grimes, Sana Hasan, Manuel Silva-Carmona, Leyat Tal, Joud Hajjar

**Affiliations:** ^1^ Division of Immunology, Allergy, and Retrovirology, Department of Pediatrics, Baylor College of Medicine, William T. Shearer Center for Human Immunobiology, Texas Children's Hospital, Houston, TX, United States; ^2^ Division of Pediatric Rheumatology, Arkansas Children's Hospital, Little Rock, AR, United States; ^3^ Division of Hematology/Oncology, Department of Pediatrics, Baylor College of Medicine, Texas Children's Hospital, Houston, TX, United States; ^4^ Division of Allergy/Immunology, Department of Medicine, Baylor College of Medicine, Houston, TX, United States; ^5^ Division of Pulmonology, Department of Pediatrics, Baylor College of Medicine, Texas Children's Hospital, Houston, TX, United States; ^6^ Division of Nephrology, Department of Pediatrics, Baylor College of Medicine, Texas Children's Hospital, Houston, TX, United States

**Keywords:** CVID, granulomatous disease, hypogammaglobulinemia, sarcoidosis, GLILD, granulomatous lymphocytic interstitial lung disease

## Abstract

Common variable immunodeficiency (CVID) can be complicated by granulomatous disease, often granulomatous lymphocytic interstitial lung disease (GLILD). Granulomatous interstitial nephritis represents an atypical presentation in pediatrics. Our patient is a previously healthy 13-year-old white male with a recent diagnosis of CVID. He presented with a rash and laboratory findings included pancytopenia (white blood cells 2.6 cells × 10^3^/μl, hemoglobin 11.8 g/dl, platelets 60 × 10^3^/μl), hypercalcemia (14.9 mg/dl), elevated Vit D 1,25 OH level (>200 pg/ml), hyperuricemia (8.8 mg/dl), and acute kidney injury (AKI) (serum creatinine 1.1 mg/dl; baseline 0.64 mg/dl). A broad infectious workup was unremarkable. The rash improved with empiric doxycycline. Hypercalcemia and hyperuricemia were managed with fluid resuscitation, calcitonin, and zoledronic acid. Evaluation for malignancy including a positron emission tomography scan, revealed multiple mediastinal hypermetabolic lymph nodes and pulmonary ground glass opacities, later reported as small pulmonary nodules by computed tomography (CT). Splenomegaly was confirmed by ultrasound and CT. Peripheral smear, bone marrow biopsy, and genetic testing were non-revealing. His angiotensin-converting enzyme level was elevated (359 U/L), raising concerns for sarcoidosis. Given Stage 1 AKI, a renal biopsy was pursued and identified non-caseating granulomatous interstitial nephritis. Treatment with 60 mg of prednisone began for presumed sarcoidosis for 4 months, causing steroid-induced hypertension and mood changes. Zoledronic acid minimally reduced serum creatinine. *Pneumocystis jirovecii* pneumonia prophylaxis was initiated due to *T*-cell cytopenia. Chest CT findings showed a suboptimal response to steroids. A bronchoalveolar lavage demonstrated >50% lymphocytes (normal <10%) and the lung biopsy exhibited non-caseating granulomas, indicating GLILD. Rubella was identified by staining. Following a fever, he was found to have elevated liver enzymes and confirmed hepatitis with portal hypertension on CT. A liver biopsy revealed epithelioid non-caseating granuloma and HHV6 was detected by PCR. He was treated with four cycles of rituximab and granulocyte-colony stimulating factor for persistent neutropenia. Subsequent treatment with mycophenolate led to the resolution of the granulomatous lesions and cytopenias. The rare complication of granulomatous interstitial nephritis in CVID illustrates the intricate nature of diagnosis. This case underscores the necessity for a holistic view of the patient’s clinical and immune phenotype, including distinctive radiological presentations, for precise diagnoses and tailored management of CVID.

## Introduction

Common variable immunodeficiency (CVID) is among the most commonly diagnosed primary immunodeficiencies (1–3). The term “variable” reflects the heterogeneous clinical presentations of patients who present with hypogammaglobulinemia and increased susceptibility to infections. The vast majority of patients lack an identifiable underlying molecular defect (4) Granulomatous disease occurs in 8%–22% of CVID patients, often affecting the lungs, lymph nodes, and spleen (5–10). Granulomatous lesions appear on organ biopsies and may precede recognition of any underlying immune defects (6–11). Resultantly, a diagnosis of CVID is often delayed and occasionally patients might be mislabeled as having sarcoidosis due to clinical overlap and the greater prevalence of sarcoidosis (5, 12). These diagnostic delays can be significantly associated with increased morbidity and mortality contributions in CVID (13). The clinical impact of non-infectious manifestations, such as granulomas, is organ-specific, and the presence of granulomas and subsequent tissue damage in the lungs and liver have been demonstrated to lead to shorter patient survival (14).

Lymphocytic interstitial infiltrate, accompanying granuloma formation, contributes to organ failure and disease severity, as seen in “granulomatous lymphocytic interstitial lung disease” or GLILD (5, 9, 15). Granuloma presence, alongside autoimmune manifestations in CVID, can significantly elevate mortality risk, particularly in pediatric-onset cases (8, 13). The development of granulomas and autoimmunity in CVID each independently pose therapeutic challenges, necessitating a careful balance of immunosuppression in an already compromised immune system. Granulomatous interstitial nephritis in adult patients with CVID has been very sparsely reported in the literature (16–18). We aim to describe a unique and rare presentation of granulomatous interstitial nephritis in a pediatric patient with known CVID. We highlight the diagnostic challenge of distinguishing CVID-associated granulomatous disease from sarcoidosis and describe the management and clinical progression of our patient, in accordance with CARE Guidelines (19).

## Narrative

Our patient was a previously healthy 13-year-old white male referred to the Allergy and Immunology service by his pediatrician following hypogammaglobulinemia identified on screening for celiac disease due to a known family history. Family history was remarkable for ulcerative colitis in the mother and an older brother with celiac disease. The patient lacked a history of recurrent or unusual infections. Immune phenotyping at the time of diagnosis (Table 1) included a complete blood count with pancytopenia [white blood cell (WBC) count 3.08 × 10^3^/μl, hemoglobin 12.5 g/dl, platelets 71 × 10^3^/μl]. He was also noted to have decreased lymphocyte subsets upon initial evaluation: CD3+ *T-*cell count 584 × 10^3^/μl, CD3+CD4+ *T-*cell count 309 × 10^3^/μl, CD3+CD8+ *T-*cell count 215 × 10^3^/μl, CD19+ B-cell count 100 × 10^3^/μl, and CD3−CD56+CD16+ NK cell count 27 × 10^3^/μl. The B-cell subset had absent class-switched memory B cells, CD19^+^CD27^+^IgM^−^IgD^−^ at 0%, and elevated transitional B-cell percentage, CD19+CD38+Bright IgM at 17% with a normal count of 16 × 10^3^/μl identified. CD19+CD38^−/low^, CD21^−/low^ autoreactive B cells were found to be 1 × 10^3^/μl. He had diffuse hypogammaglobulinemia with IgG 246 mg/dl, IgA 18 mg/dl, and IgM 7 mg/dl. Pneumococcal IgG antibodies were positive in only 1 out of 23 serotypes, with a level >1.3 μg/ml. The patient was up to date on his childhood vaccines.

**Table 1:** Laboratory studies at diagnosis.

Complete blood count	Patient's value	Normal value
White blood cell count	**3.08 × 10^3^/μl**	4.5–13.5 × 10^3^/μl
Hemoglobin	**12.5 g/dl**	13.0–16.0 g/dl
Platelets	**71 × 10^3^/μl**	150–450 × 10^3 ^μl
Immunophenotyping		
CD3^+^ cells × 10^3^/ml (%)	**584 × 10^3^/μl** (82)	1,000–2,200 × 10^3^/μl (60–76)
CD4^+^ T cells × 10^3^/ml (%)	**309 × 10^3^/μl** (43)	530–1,300 × 10^3^/μl (31–52)
CD8^+^ T cells × 10^3^/ml (%)	**215 × 10^3^/μl** (30)	330–920 × 10^3^ (18–35)
CD4^+^/CD8^+^ *T*-cell ratio	1.44	0.7–2.4
CD19^+^ B cells × 10^3^/ml (%)	**100 × 10^3^/μl** (14)	110–570 × 10^3^/μl (6–23)
CD27^+^ Memory B cells × 10^3^/ml (%)	3 × 10^3^/μl (4)	2–122 × 10^3^/μl (2–36)
CD19^+^CD27^+^IgM^−^IgD^−^ Class-Switched Memory B cells × 10^3^/ml (%)	0 × 10^3^/μl (0)	0–74 × 10^3^/μl (0–22)
CD19^+^CD38^+^Bright IgM^+^ Transitional B cells × 10^3^/ml (%)	16 × 10^3^/μl (17)	0–18 × 10^3^/μl (0–6)
CD56^+^ CD16^+^ NK cells × 10^3^/ml (%)	**27 × 10^3^/μl** (4)	70–480 × 10^3^/μl (3–22)
CD19^+^CD38^−/low^ CD21^−/low^ autoreactive B cells	1 × 10^3^ (1)	0–21 × 10^3^/μl (0%–7%)
Mitogenic and antigenic induced lymphocyte proliferation		
Phytohemagglutinin 10 μg/ml	**40, 826 lymphocyte proliferation in counts per minute**	>163,507 Lymphocyte proliferation in counts per minute
Tetanus antigen	4,340 Lymphocyte proliferation in counts per minute	>2,000 lymphocyte proliferation in counts per minute
Cytokines		
Soluble IL-2 receptor (CD25)	**8,711 U/ml**	45–1,105 unit/ml
Immunoglobulins		
IgG	246 mg/dl	641–1,353 mg/dl
IgA	18 mg/dl	66–295 mg/dl
IgM	7 mg/dl	40–80 mg/dl
Vaccination antigen response		
Pneumococcal IgG	1/23 > 1.3 μg/dl	50%–70% > 1.3 μg/dl
*Haemophilus influenzae* B IgG	<0.15 μg/ml	≥1.00 μg/ml
Tetanus antitoxoid	0.10 IU/ml	≥0.10 IU/ml

Bold values represent those values outside of the listed reference ranges.

Shortly after the initial immune evaluation, our patient developed a diffuse non-blanching maculopapular rash and progression of pancytopenia (WBC 2.66 × 10^3^/μl, Hemoglobin 11.8 g/dl, Platelet 60 × 10^3^/μl) following a camping trip in the Ozarks, an area endemic for several tick-borne illnesses, which occurred 1 month before the rash onset. His rash was non-pruritic, noted to involve the palms and soles, and appeared petechial in nature around his thighs and axilla. Screening labs on admission displayed hypercalcemia (14.9 mg/dl), elevated Vitamin D 1,25 OH level (>200 pg/ml), hyperuricemia (8.8 mg/dl), and acute kidney injury (AKI) (serum creatinine 1.1 mg/dl up from baseline 0.64 mg/dl). Soluble IL-2 was elevated to a peak of 8,711 U/ml, with ratio of soluble IL-2 to WBC of 3.5.

**Figure 1:**
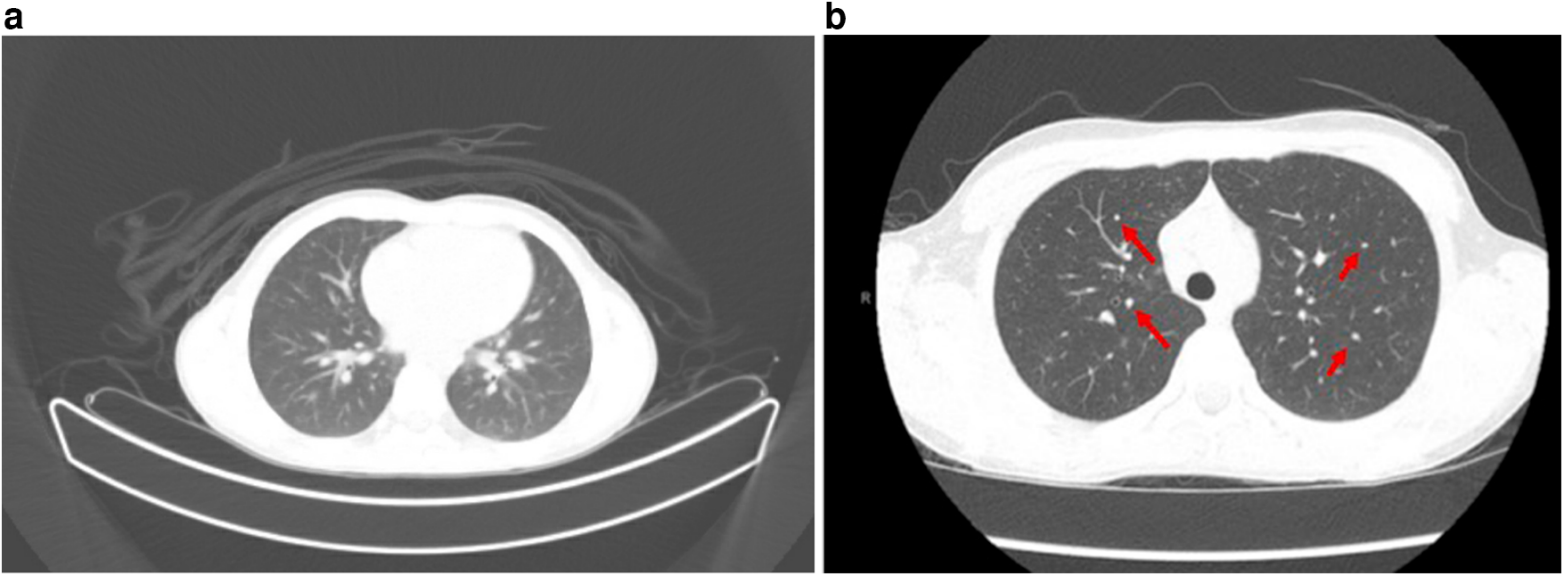
**(a)** PET scan with multiple mediastinal, axillary, and common hilar lymph nodes are appreciated, with representative lymph nodes. Multiple ground glass nodules within the bilateral lungs predominantly in a centrilobular distribution. Additional indistinct ground glass opacities along the posterior aspect of the bilateral lung. **(b)** CT scan with enlarged mediastinal lymph nodes demonstrated, correlating with hypermetabolic nodes from PET above. Multiple non-specific subcentimeter ground glass nodules (red arrows) throughout both lungs.

The constellation of pancytopenia, hypercalcemia, and hyperuricemia triggered an evaluation for malignancy. The positron emission tomography (PET) (Figure 1a) scan showed multiple hypermetabolic lymph nodes within his mediastinum nodal enlargement and pulmonary ground glass opacities. Computed tomography (CT) (Figure 1b) later reported pulmonary changes as diffuse small pulmonary nodules. Splenomegaly was confirmed by ultrasound (US) and CT, and left kidney enlargement was seen on the US. Peripheral smear and bone marrow biopsy findings were normal. Concurrent broad evaluation for infection, including tick-borne illnesses, was pursued and was unremarkable. A critical trio whole-exome sequencing was also unrevealing. He was started on a 10-day empiric course of doxycycline with an improvement in the rash. Hypercalcemia and hyperuricemia were managed with fluid resuscitation, calcitonin, and zoledronic acid. He received intravenous IgG replacement (500 mg/kg) during admission and continued monthly replacement with a goal trough of 1,000 mg/ml.

The presence of hypercalcemia, elevated Vitamin D levels, hypermetabolic lymph nodes, and pulmonary manifestations prompted an assessment for lymphoproliferative disorder. A renal biopsy was obtained due to Stage 1 AKI and identified non-caseating granuloma on electron microscopy, raising concern for sarcoidosis. Hematoxylin and eosin staining revealed numerous well-formed epithelioid, non-caseating granulomas with focal extension into renal tubules, in addition to the presence of focal calcified bodies and Langerhans-type giant cells. His angiotensin-converting enzyme (ACE) level was found to be elevated at 359 U/L (reference range 13–100 U/L), and universal polymerase chain reaction (PCR) testing on the renal biopsy was found to be negative for acid-fast bacilli, bacterial, and fungal infection.

**Figure 2:**
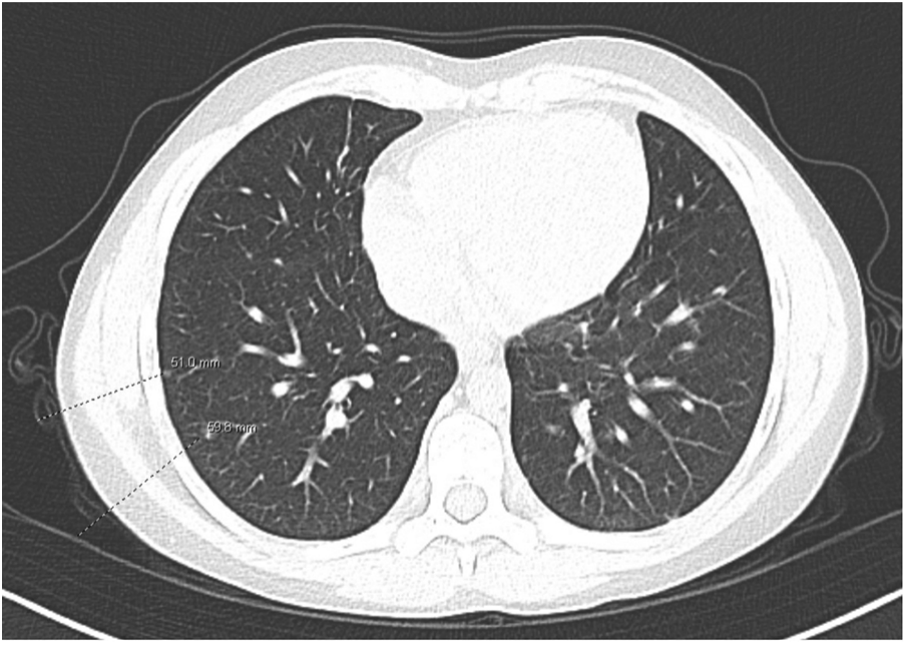
CT following the use of steroids. Mixed response in multiple pulmonary nodules, with some nodules having resolved and other nodules new.

A course of prednisone 60 mg daily for presumed sarcoidosis was initiated for a 4-month period with a slow taper. While on steroids, he developed side effects including steroid-induced hypertension and mood changes, in addition to a suboptimal clinical response. He also remained cytopenic while on steroid therapy and required *Pneumocystis jirovecii* pneumonia prophylaxis for persistent *T*-cell lymphopenia. Chest CT findings also showed an almost negligible response to steroids; though some nodules were resolving, there was formation of new nodules (Figure 2). A bronchoalveolar lavage had greater than 50% lymphocytes (normal <10%). A lung biopsy was pursued to further characterize the lung nodules identified on CT to dictate the next therapeutic steps and it confirmed the presence of multi-organ granulomatous disease with the presence of non-caseating granulomas and lymphocytic inflammation, suggesting a diagnosis of GLILD.

Shortly after the biopsy, he spiked a fever and was found to have elevated liver enzymes. CT and ultrasound findings confirmed hepatitis with a suspicion of portal hypertension. A liver biopsy was performed and revealed epithelioid granulomas without necrotizing or caseating features, with the presence of focal areas of lobular and portal chronic inflammation. Histochemical staining identified focal glycogenated nuclei without copper or iron accumulation. Human herpes virus (HHV6) was detected in liver tissue by PCR.

He was treated with four cycles of rituximab, which normalized hemoglobin and platelet counts; however, he remained persistently neutropenic. Resultantly, he was started on granulocyte-colony stimulating factor (G-CSF) 5 μg/kg/day, which led to an improvement in absolute neutrophil counts. He subsequently received treatment with mycophenolate (MMF) for immune-mediated cytopenias, in addition to his multi-organ granulomatous disease, with good response, allowing discontinuation of G-CSF. Due to a lack of steroids and the patient's intolerance to side effects, no further steroids were administered. Our patient demonstrated improvements in white blood cell, hemoglobin, and platelet counts. While he remained *T*-cell lymphopenic, he maintained normal lymphocyte proliferation, obviating the need for *Pneumocystis jirovecii* prophylaxis. Following 2 years of therapy, he experienced complete resolution of pulmonary ground glass opacities and nodules, and hepatosplenomegaly.

## Discussion

Granulomatous findings in the tissue of patients with CVID have been noted to be mistaken for sarcoidosis (7, 12). Discriminating between these conditions can be complex due to overlapping clinical and diagnostic features, including multi-system non-caseating granulomas with hypercalcemia, lymphadenopathy, and propensity for pulmonary, liver, and lymph node involvement (20, 21). In some situations, arriving at an accurate diagnosis can be further complicated by an immune phenotype not yet identified.

Elucidating the differences between granulomatous disease in CVID and sarcoidosis is critically important to avoid inappropriate treatment and delays in targeted treatment (Table 2). Many patients with sarcoidosis in fact have normal ACE levels (7, 22, 23). ACE levels have also been reported to be abnormally elevated in CVID patients without granulomas (8). Further, ACE levels can be elevated in the setting of certain infections such as the human immunodeficiency virus (HIV) and other infections presenting with non-caseating granulomas such as histoplasmosis (24, 25). Both sarcoidosis and granulomatous disease in CVID include B-cell derangements; however, CVID can be delineated by the absence or the reduction of switched memory B cells, and sarcoidosis favors reduced memory B cells alone (20). Sarcoidosis is typically characterized by the presence of hypergammaglobulinemia rather than hypogammaglobulinemia, a hallmark of CVID (26). Notably, patients with CVID and GLILD display markers reflecting *T*-cell activation and exhaustion, including soluble IL-2 receptor when compared to other patients with CVID with non-infectious complications (27).

**Table 2:** Key comparisons in CVID with GLILD and sarcoidosis.

Feature	CVID with GLILD	Sarcoidosis
B-cell subset	Absent or reduced memory switched B cells	Reduced memory B cells
Immunoglobulins	Hypogammaglobulinemia	Hypergammaglobulinemia or normal
Lung granulomas	Present	Present
Lymph node biopsy histologic findings	Ill-defined germinal centers, decreased plasma cells	Undisrupted architecture
Chest CT findings	Lower lung lobe with larger nodularity, ground glass opacities, and flame-shaped hemorrhages	Perilymphatic micronodular infiltrate in bronchovascular distribution
Bronchoalveolar lavage fluid	Normal CD4:CD8 (<3:1)	Elevated CD4:CD8 ratio (>3:5)
ACE levels	Normal or elevated	Normal or elevated
Spontaneous remission	Rare	Common in early stages

From a histopathologic standpoint, lymphoid hyperplasia has been observed alongside granulomas in CVID and is absent in sarcoidosis. Specifically, patients with GLILD have exhibited CD4+ *T*-cell predominance in the lungs, variable CD8+ *T* cells and B cells were found in much smaller numbers. A notable absence of regulatory T cells in lung biopsies has also been reported (28). Follicular helper *T* cells have been noted within granuloma in CVID, whereas they are more weakly expressed in sarcoidosis (29). The presence of organizing pneumonias has been found in GLILD and a subgroup of patients with CVID and GLILD will progress to interstitial fibrosis with architectural remodeling (28).

Lymph node biopsy in CVID patients usually demonstrates disrupted architecture with ill-defined germinal centers and marked reductions in plasma cells, whereas plasma cells and germinal cells are more likely to be intact in sarcoidosis (20, 30). High-resolution CT and bronchoalveolar lavage findings can also help discriminate these conditions (12). GLILD on chest CT is more likely to include lower lobe disease with larger nodularity, ground glass opacities, and flame-shaped hemorrhages, while sarcoidosis is more often associated with perilymphatic micronodular infiltrate appearing in a bronchovascular distribution (31, 32). In the bronchoalveolar fluid, a CD4:CD8 ratio is typically normal in CVID and elevated (>3.5) in sarcoidosis (32).

It is estimated that 2% of patients with CVID have renal insufficiency (33). One study indicated that membranous glomerulonephropathy and tubulointerstitial nephritis were the predominant pathologic findings in renal biopsies from CVID patients with AKI and/or proteinuria (34).

Interstitial lymphocytic infiltration, in conjunction with granulomatous formations, indicates an increased risk of organ failure and disease severity (5). While up to 22% of patients with CVID have some variation of granulomatous disease, this prevalence is likely under-representative as most patients do not undergo routine biopsies (2, 35, 36). Tissue biopsy is not only crucial for diagnosis of granulomas but also to distinguish it from other conditions such as lymphoid hyperplasia and lymphomas (37, 38).

**Figure 3:**
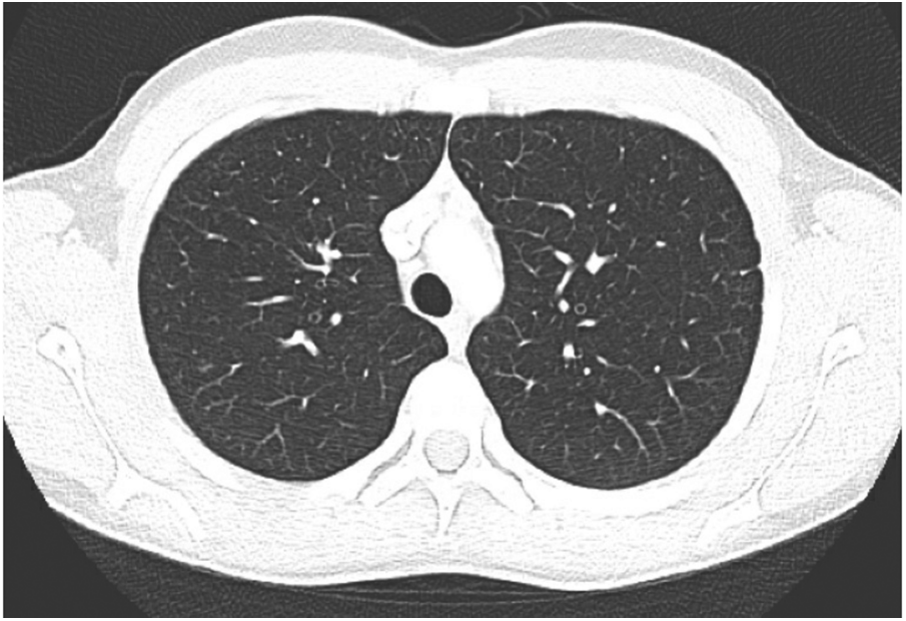
CT scan demonstrating interval near resolution of previously described punctate nodules throughout both lungs. Two isolated ground glass foci remain with the right lung. No new nodule or ground glass opacity.

Our patient developed an exceedingly rare complication of granulomatous interstitial nephritis in CVID, in addition to GLILD, liver granulomas, and cytopenias. Initially, a diagnosis of sarcoidosis was made, however, his clinical presentation and features were more consistent with the granulomatous manifestations known to occur in CVID. An elevated soluble IL-2 receptor to white blood cell ratio, as was present in our patient, has been demonstrated to reflect granulomatous disease progression in CVID and may represent an important and readily available biomarker for risk stratification for this disease population (39). He clinically responded to a tailored treatment regimen with four cycles of rituximab, G-CSF, and mycophenolate for persistent neutropenia with resolution of the granulomas (Figure 3) and cytopenias.

## Conclusion

This case underscores the diagnostic complexities inherent in differentiating CVID-related granulomatous disease from other similar conditions. The coexistence of hypogammaglobulinemia with specific chest CT findings steered the diagnosis toward GLILD rather than sarcoidosis. This case emphasizes the necessity of a thorough evaluation of a patient's clinical presentation and immune phenotyping when CVID-related granulomatous anomalies are suspected. Meticulous attention to the distinctive clinical and diagnostic features is paramount for achieving an accurate diagnosis, which is crucial for the effective management and targeted treatment of patients with CVID.

## Data Availability

The original contributions presented in the study are included in the article/Supplementary Material, further inquiries can be directed to amanda.salih@alumni.bcm.edu.

## References

[1] Gathmann B Goldacker S Klima M Belohradsky BH Notheis G Ehl S The German national registry for primary immunodeficiencies (PID). Clin Exp Immunol. (2013) 173(2):372–80. 10.1111/cei.12105 23607573 PMC3722937

[2] Gathmann B Mahlaoui N Ceredih GL Oksenhendler E Warnatz K Schulze I Clinical picture and treatment of 2212 patients with common variable immunodeficiency. J Allergy Clin Immunol. (2014) 134(1):116–26. 10.1016/j.jaci.2013.12.1077 24582312

[3] Sullivan KE Puck JM Notarangelo LD Fuleihan R Caulder T Wang C USIDNET: a strategy to build a community of clinical immunologists. J Clin Immunol. (2014) 34(4):428–35. 10.1007/s10875-014-0028-1 24711005 PMC4046905

[4] Yang Y Muzny DM Xia F Niu Z Person R Ding Y Molecular findings among patients referred for clinical whole-exome sequencing. JAMA. (2014) 312(18):1870–9. 10.1001/jama.2014.14601 25326635 PMC4326249

[5] Ardeniz O Cunningham-Rundles C. Granulomatous disease in common variable immunodeficiency. Clin Immunol. (2009) 133(2):198–207. 10.1016/j.clim.2009.05.001 19716342 PMC2760682

[6] Cunningham-Rundles C. Common variable immunodeficiency. Curr Allergy Asthma Rep. (2001) 1(5):421–9. 10.1007/s11882-001-0027-1 11892068

[7] Fasano MB Sullivan KE Sarpong SB Wood RA Jones SM Johns CJ Sarcoidosis and common variable immunodeficiency. Report of 8 cases and review of the literature. Medicine (Baltimore). (1996) 75(5):251–61. 10.1097/00005792-199609000-00002 8862347

[8] Mechanic LJ Dikman S Cunningham-Rundles C. Granulomatous disease in common variable immunodeficiency. Ann Intern Med. (1997) 127(8 Pt 1):613–7. 10.7326/0003-4819-127-8_Part_1-199710150-00005 9341059

[9] Morimoto Y Routes JM. Granulomatous disease in common variable immunodeficiency. Curr Allergy Asthma Rep. (2005) 5(5):370–5. 10.1007/s11882-005-0008-x 16091208

[10] Mullighan CG Fanning GC Chapel HM Welsh KI. TNF and lymphotoxin-alpha polymorphisms associated with common variable immunodeficiency: role in the pathogenesis of granulomatous disease. J Immunol. (1997) 159(12):6236–41. 10.4049/jimmunol.159.12.6236 9550427

[11] Bonilla FA Barlan I Chapel H Costa-Carvalho BT Cunningham-Rundles C de la Morena MT International consensus document (ICON): common variable immunodeficiency disorders. J Allergy Clin Immunol Pract. (2016) 4(1):38–59. 10.1016/j.jaip.2015.07.025 26563668 PMC4869529

[12] Verbsky JW Routes JM. Sarcoidosis and common variable immunodeficiency: similarities and differences. Semin Respir Crit Care Med. (2014) 35(3):330–5. 10.1055/s-0034-1376862 25007085

[13] Baloh C Reddy A Henson M Prince K Buckley R Lugar P. 30-year review of pediatric- and adult-onset CVID: clinical correlates and prognostic indicators. J Clin Immunol. (2019) 39(7):678–87. 10.1007/s10875-019-00674-9 31377970 PMC6754754

[14] Ho HE Cunningham-Rundles C. Non-infectious complications of common variable immunodeficiency: updated clinical spectrum, sequelae, and insights to pathogenesis. Front Immunol. (2020) 11:149. 10.3389/fimmu.2020.00149 32117289 PMC7025475

[15] Bates CA Ellison MC Lynch DA Cool CD Brown KK Routes JM. Granulomatous-lymphocytic lung disease shortens survival in common variable immunodeficiency. J Allergy Clin Immunol. (2004) 114(2):415–21. 10.1016/j.jaci.2004.05.057 15316526

[16] Meyer A Lachmann HJ Webster AD Burns A Thway K. Hypercalcemia in a patient with common variable immunodeficiency and renal granulomas. Am J Kidney Dis. (2005) 45(5):e90–3. 10.1053/j.ajkd.2005.02.023 15861342

[17] Stigant C Sapir D Sweet J Downey G Bargman JM. A unique renal lesion in common variable immunodeficiency. Clin Nephrol. (2002) 57(1):74–9. 10.5414/CNP57074 11837805

[18] Fakhouri F Robino C Lemaire M Droz D Noel LH Knebelmann B Granulomatous renal disease in a patient with common variable immunodeficiency. Am J Kidney Dis. (2001) 38(2):E7. 10.1053/ajkd.2001.26095 11479181

[19] Riley DS Barber MS Kienle GS Aronson JK von Schoen-Angerer T Tugwell P CARE guidelines for case reports: explanation and elaboration document. J Clin Epidemiol. (2017) 89:218–35. 10.1016/j.jclinepi.2017.04.026 28529185

[20] Ameratunga R Ahn Y Tse D Woon ST Pereira J McCarthy S The critical role of histology in distinguishing sarcoidosis from common variable immunodeficiency disorder (CVID) in a patient with hypogammaglobulinemia. Allergy Asthma Clin Immunol. (2019) 15:78. 10.1186/s13223-019-0383-9 31827542 PMC6886192

[21] Jain R Yadav D Puranik N Guleria R Jin JO. Sarcoidosis: causes, diagnosis, clinical features, and treatments. J Clin Med. (2020) 9(4):1081. 10.3390/jcm9041081 PMC7230978 32290254

[22] Ungprasert P Carmona EM Crowson CS Matteson EL. Diagnostic utility of angiotensin-converting enzyme in sarcoidosis: a population-based study. Lung. (2016) 194(1):91–5. 10.1007/s00408-015-9826-3 26563332 PMC4768304

[23] Zheng SY Du X Dong JZ. Re-evaluating serum angiotensin-converting enzyme in sarcoidosis. Front Immunol. (2023) 14:950095. 10.3389/fimmu.2023.950095 37868968 PMC10586325

[24] Ouellette DR Kelly JW Anders GT. Serum angiotensin-converting enzyme level is elevated in patients with human immunodeficiency virus infection. Arch Intern Med. (1992) 152(2):321–4. 10.1001/archinte.1992.00400140069016 1310846

[25] Narula N Iannuzzi M. Sarcoidosis: pitfalls and challenging mimickers. Front Med (Lausanne). (2020) 7:594275. 10.3389/fmed.2020.594275 33505980 PMC7829200

[26] Hunninghake GW Crystal RG. Mechanisms of hypergammaglobulinemia in pulmonary sarcoidosis. Site of increased antibody production and role of T lymphocytes. J Clin Invest. (1981) 67(1):86–92. 10.1172/JCI110036 6969734 PMC371575

[27] Fraz MSA Michelsen AE Moe N Aalokken TM Macpherson ME Nordoy I Raised serum markers of T cell activation and exhaustion in granulomatous-lymphocytic interstitial lung disease in common variable immunodeficiency. J Clin Immunol. (2022) 42(7):1553–63. 10.1007/s10875-022-01318-1 35789314 PMC9255534

[28] Rao N Mackinnon AC Routes JM. Granulomatous and lymphocytic interstitial lung disease: a spectrum of pulmonary histopathologic lesions in common variable immunodeficiency—histologic and immunohistochemical analyses of 16 cases. Hum Pathol. (2015) 46(9):1306–14. 10.1016/j.humpath.2015.05.011 26138782 PMC4554947

[29] Viallard JF Lescure M Oksenhendler E Blanco P Visentin J Parrens M. STAT expression and TFH1 cells in CVID granulomatosis and sarcoidosis: immunological and histopathological comparisons. Virchows Arch. (2024) 484(3):481–90. 10.1007/s00428-023-03684-6 37924346

[30] Unger S Seidl M Schmitt-Graeff A Bohm J Schrenk K Wehr C Ill-defined germinal centers and severely reduced plasma cells are histological hallmarks of lymphadenopathy in patients with common variable immunodeficiency. J Clin Immunol. (2014) 34(6):615–26. 10.1007/s10875-014-0052-1 24789743

[31] Rodriguez JA Bang TJ Restrepo CS Green DB Browne LP Vargas D. Imaging features of primary immunodeficiency disorders. Radiol Cardiothorac Imaging. (2021) 3(2):e200418. 10.1148/ryct.2021200418 33969305 PMC8098094

[32] Perlman DM Sudheendra MT Racilla E Allen TL Joshi A Bhargava M. Granulomatous-lymphocytic interstitial lung disease mimicking sarcoidosis. Sarcoidosis Vasc Diffuse Lung Dis. (2021) 38(3):e2021025. 10.36141/svdld.v38i3.11114 34744421 PMC8552568

[33] Hermaszewski RA Webster AD. Primary hypogammaglobulinaemia: a survey of clinical manifestations and complications. Q J Med. (1993) 86(1):31–42. 8438047

[34] Caza TN Hassen SI Larsen CP. Renal manifestations of common variable immunodeficiency. Kidney360. (2020) 1(6):491–500. 10.34067/KID.0000432020 35368588 PMC8809320

[35] Cunningham-Rundles C. Common variable immune deficiency: dissection of the variable. Immunol Rev. (2019) 287(1):145–61. 10.1111/imr.12728 30565247 PMC6435035

[36] Boursiquot JN Gerard L Malphettes M Fieschi C Galicier L Boutboul D Granulomatous disease in CVID: retrospective analysis of clinical characteristics and treatment efficacy in a cohort of 59 patients. J Clin Immunol. (2013) 33(1):84–95. 10.1007/s10875-012-9778-9 22986767

[37] Maglione PJ Ko HM Beasley MB Strauchen JA Cunningham-Rundles C. Tertiary lymphoid neogenesis is a component of pulmonary lymphoid hyperplasia in patients with common variable immunodeficiency. J Allergy Clin Immunol. (2014) 133(2):535–42. 10.1016/j.jaci.2013.08.022 24131823 PMC4109033

[38] Chapel H Lucas M Lee M Bjorkander J Webster D Grimbacher B Common variable immunodeficiency disorders: division into distinct clinical phenotypes. Blood. (2008) 112(2):277–86. 10.1182/blood-2007-11-124545 18319398

[39] van Stigt AC Dalm V Nagtzaam NMA van Hagen PM Dik WA IJspeert H. Soluble interleukin-2 receptor/white blood cell ratio reflects granulomatous disease progression in common variable immune deficiency. J Clin Immunol. (2023) 43(8):1754–7. 10.1007/s10875-023-01560-1 37542638 PMC10661782

